# Hyperuricemia is associated with atrial fibrillation prevalence in very elderly - a community based study in Chengdu, China

**DOI:** 10.1038/s41598-018-30321-z

**Published:** 2018-08-17

**Authors:** Gang Huang, Rong-hua Xu, Jun-bo Xu, Ya liu, Zhao-hui Liu, Xue Xie, Ting-jie Zhang

**Affiliations:** 1 Cardiovascular and Metabolic Disease Center, The Second People’s Hospital of Chengdu, Chengdu, China; 2 Department of Cardiology, The Second People’s Hospital of Chengdu, Chengdu, China

## Abstract

Hyperuricemia is a risk factor for cardiovascular diseases. However, in very elderly, the relationship between hyperuricemia and the prevalence of atrial fibrillation (AF) is not clear. This study aimed to investigate hyperuricemia and the risk of AF in community very elderly. In this cross-sectional study, 1056 very elderly in community were enrolled. Serum uric acid (SUA) were measured and rest 12-lead electrocardiogram was performed. Multiple logistic regression models were used to explore risk factors for AF in very elderly. Finally, 1038 participants were included in analysis and the mean age of the study participants were 83.6 ± 3.4 years (age range 80–100 years). The mean SUA level was 350.1 ± 84.5 µmol/L. The estimated prevalence of AF was 5.3%, and there was no significant sex difference (5.8% for men and 4.8% for women, p = 0.401). Multiple logistic regression found that participants with hyperuricemia (SUA >416 µmol/L in men and >357 µmol/L in women) had a higher risk (odds ratio: 2.080, 95% confidence interval: 1.103–4.202, P = 0.007) of suffering AF in very elderly Chinese. In conclusion, AF is relatively frequent in this community very elderly Chinese in Chengdu. Hyperuricemia is associated with the prevalence of AF in general very elderly.

## Introduction

Atrial fibrillation (AF) is the most common cardiac arrhythmia and an important cause of cardiovascular morbidity and mortality worldwide^1^. It is already known that the prevalence and incidence of AF increase with aging and they are higher in men than in women, and the prevalence of AF is lower in Chinese than in Caucasian^2^. Approximately 8 million Chinese adults suffer from AF^2^. Moreover, AF accounts for a 4 to 5-fold increased risk of stroke and is responsible for approximately 15% of all strokes in the United States^3^.

As the end product of purine metabolism in humans, serum uric acid (SUA) is an independent marker of morbidity and mortality in a variety of cardiovascular diseases^4,5^. It has been reported that hyperuricemia is associated with many cardiovascular diseases, including heart failure, hypertension, ischemic heart disease, and stroke^5–8^.

Although previous studies have reported that hyperuricemia is positively associated with AF in patients with different diseases, less is known in the community general population, especially in the very elderly^9–11^. Especially, very elderly were not pre specified as study subjects. Therefore, this study aimed to describe the rest ECG based AF prevalence in community very elderly Chinese in Chengdu and investigate the association between SUA and AF.

## Results

### Characteristics of Study Population

Of the 1056 very elderly participants, 18 participants were excluded because of incomplete data. Therefore, 1038 participants with a mean age of 83.6 ± 3.4 years were enrolled in final analysis, 49.8% of which were men. Participants with AF (n = 55) were older and had significantly higher SUA level, diastolic BP and heart rate than those without AF (all Ps <0.05). However, participants with AF had significantly lower levels of TC, TG, LDL-C and pulse pressure (PP) (all Ps <0.05) (Table 1).

**Table 1:** Characteristics of Participants.

	Total n = 1038	AF n = 55	Non AF n = 983	p value
Age (years)	83.6 ± 3.4	83.9 ± 3.6	83.6 ± 3.4	0.552
Current smoker, %(n)	11.1 (115)	5.5 (3)	11.4 (112)	0.173
Current drinker, %(n)	8.3 (86)	14.5 (8)	8.0 (78)	0.125
Medical history, %(n)				
Hypertension	52.6 (546)	56.4 (31)	52.4 (515)	0.566
DM	12.3 (128)	9.1 (5)	12.5 (123)	0.453
Stroke	8.8 (91)	5.5 (3)	9.0 (88)	0.470
MI	7.2 (75)	7.3 (4)	7.2 (71)	0.989
Medication %(n)				
ACEI/ARB	11.9 (124)	12.7 (7)	11.9 (117)	0.854
CCB	26.1 (271)	34.5 (19)	25.6 (252)	0.143
β-receptor blocker	7.8 (81)	20.0 (11)	7.1 (70)	0.002
Diuretics	6.6 (68)	3.6 (2)	6.7 (66)	0.574
BMI (kg/m^2^)	23.1 ± 3.7	23.7 ± 4.1	23.1 ± 3.7	0.282
SBP (mmHg)	146.4 ± 20.6	145.4 ± 21.0	146.6 ± 22.4	0.830
DBP (mmHg)	74.1 ± 11.9	82.5 ± 14.9	73.7 ± 11.7	<0.001
PP (mmHg)	72.5 ± 17.1	63.9 ± 16.1	73.6 ± 18.2	<0.001
Heart rate (bpm)	70.0 ± 9.0	72.0 ± 14.9	69.5 ± 7.6	0.005
FBG (mmol/L)	5.53 ± 1.35	5.37 ± 1.19	5.55 ± 1.38	0.081
TC (mmol/L)	4.87 ± 0.99	4.46 ± 0.97	4.89 ± 0.98	0.001
TG (mmol/L)	1.34 ± 0.57	1.15 ± 0.51	1.37 ± 0.80	0.020
LDL-C (mmol/L)	2.58 ± 0.74	2.27 ± 0.70	2.60 ± 0.74	0.001
SUA (µmol/L)	350.1 ± 84.5	382.3 ± 94.0	348.9 ± 89.6	0.005
Creatinine, μmol/L	104.1 ± 32.4	110.2 ± 27.8	103.9 ± 33.0	0.008
e GFR, ml/(min∙1.73 m^2^)	58.7 ± 13.9	55.3 ± 13.5	58.6 ± 13.9	0.084

Data are presented as mean ± standard deviation or Percentage(number). P value from comparison between AF group and non AF group. ACEI indicates angiotensin-converting enzyme inhibitor; AF, atrial fibrillation; ARB, angiotensin receptor blocker; BMI, body mass index; CCB, calcium channel blockers; COPD, chronic obstructive pulmonary disease; DBP, diastolic blood pressure; eGFR, estimated glomerular filtration rate, FBG, fasting blood glucose; LDL-C, Low-density lipoprotein cholesterol; MI, myocardial infarction; PP, pulse pressure; SBP, systolic blood pressure; SUA, serum uric acid; TC, total cholesterol; TG, triglycerides. eGFR was calculated according to Cockcroft-Gault equation.

### SUA level and prevalence of Hyperuricemia

The mean level of SUA in overall participants was 350.1 ± 84.5 µmol/L. Participants with AF had a significantly higher SUA level (382.3 ± 94.0 µmol/L vs 348.9 ± 89.6 µmol/L, p = 0.005). The estimated prevalence of hyperuricemia was 33.0% (n = 342) and there was no sex difference (30.2% for men and 35.6% for women, p = 0.075).

### Estimated Prevalence of AF

The variability of ECG analysis was about 3.0% (31/1038). The overall estimated prevalence of AF was 5.3% in our study population. And there was no sex difference in prevalence of AF (5.8% for men and 4.8% for women, p = 0.401). The overall AF prevalence was the highest in participants aged from 85 to 89 year. Men aged older than 90 years had the highest prevalence, while women aged 85 to 89 year had the highest prevalence of AF (Table 2).

**Table 2:** Prevalence of AF.

	Prevalence of AF, %(n)			
	Total	Men	Women	p value
Total	5.3 (55)	5.8 (30)	4.8 (25)	0.401
Age 80–84	4.7 (34)	5.8 (21)	3.6 (13)	0.146
85–89	6.9 (17)	5.6 (7)	8.2 (10)	0.552
≥90	5.8 (4)	6.3 (2)	5.4 (2)	0.976
Participants with				
Hypertension	5.9 (47)	7.2 (28)	4.7 (19)	0.137
New hypertension	6.0 (15)	7.7 (10)	4.1 (5)	0.234
Self reported hypertension	5.9 (32)	6.9 (18)	4.9 (14)	0.324
Prehypertension	2.9 (5)	2.4 (2)	3.3 (3)	0.717
Normotension	4.4 (3)	4.7 (2)	4.0 (1)	0.900
DM	5.7 (12)	6.3 (7)	5.1 (5)	0.709
New DM	8.6 (5)	10.8 (4)	5.6 (1)	0.658
Self reported DM	4.6 (7)	4.1 (3)	5.0 (4)	0.778
IFG	3.0 (2)	5.1 (2)	0	0.509
NFG	5.4 (41)	6.3 (23)	4.5 (18)	0.292
Obesity	10.3 (10)	14.6 (6)	7.1 (4)	0.314
Non-obesity	4.8 (45)	5.5 (26)	4.1 (19)	0.323
Hyperuricemia	7.6 (26)	10.3 (16)	5.4 (10)	0.108
Non-hyperuricemia	4.2 (29)	4.4 (16)	3.9 (13)	0.716

Percentages represent the number with atrial fibrillation/total population. P value are from comparison between men and women. AF indicates atrial fibrillation; DM, diabetes mellitus; IFG, impaired fasting glucose; NFG, normal fasting glucose.

Overall AF prevalence in participants with hyperuricemia, or with obesity, or with hypertension and with DM was 10.3%, 7.6%, 5.9% and 5.7%, respectively. In addition, AF prevalence in participants with newly diagnosed hypertension or DM was relatively higher, compared with which in self reported hypertensive participants or diabetic participants (p = 0.949 for comparison between newly diagnosed and self reported hypertension, p = 0.245, for comparison between newly diagnosed and self reported DM). As well, AF prevalence in participants with hyperuricemia (p = 0.020, compared with non hyperuricemia participants) or obesity (p < 0.001, compared with non obesity) was significantly higher (Table 2).

### Hyperuricemia and AF

In multiple logistic regression model, after adjustment for age, sex, smoking, obesity, hypertenson, DM, PP, TG, TC, and e GFR, hyperuricemia was an independent factor for AF in overall participants (odds ratio (OR): 2.080, 95%CI: 1.103–4.202, P = 0.007, Table 3), in participants with hypertension (OR: 2.452, 95%CI: 1.230–5.238, P = 0.002) or patients with DM (OR: 10.254, 95%CI: 1.350–67.137, P = 0.020) after adjustment for factors above, hyperuricemia was also a factor for AF (Table 3). When SUA was included as continuous variable, it is also an independent factor for AF in overall participants (OR: 1.521, 95%CI: 1.256–5.754, P = 0.003) in participants with hypertension (OR: 1.736, 95%CI: 1.131–6.574, P = 0.030) or patients with DM (OR: 1.106, 95%CI: 1.099–69.035, P = 0.045).

**Table 3:** Multiple logistic regression analysis of risk factors for AF in total and subgroups.

	Total	Hypertension	DM
Age (years)			
80–84	Ref.	Ref.	Ref.
85–89	1.971 (0.995–3.860)	2.842 (1.363–6.573)*	3.062 (0.484–20.013)
≥90	2.026 (0.646–6.248)	4.153 (1.148–13.462)*	0.000
Sex			
Men	Ref.	Ref.	Ref.
Women	0.706 (0.357–1.270)	0.701 (0.335–1.417)	0.524 (0.101–3.362)
Smoking			
No	Ref.	Ref.	Ref.
Yes	0.344 (0.102–1.465)	0.421 (0.11–1.825)	1.040 (0.072–17.234)
Obesity			
No	Ref.	Ref.	Ref.
Yes	2.502 (1.114–6.041)*	3.032 (1.115–6.371)*	3.53 (0.392–30.348)
Hypertension			
No	Ref.	—	Ref.
Yes	3.637 (1.385–9.325)*		3.317 (0.279–45.436)
PP (mmHg)			
<60	Ref.	Ref.	Ref.
60–79	0.301 (0.125–0.652)*	0.232 (0.108–0.552)*	0.503 (0.084–3.013)
≥80	0.115 (0.047–0.314)*	0.101 (0.041–0.266)*	0.000
DM			
No	Ref.	Ref.	—
Yes	0.725 (0.323–1.561)	0.716 (0.301–1.622)	
Hyperuricemia			
No	Ref.	Ref.	Ref.
Yes	2.080 (1.103–4.202)*	2.452 (1.230–5.238)*	10.254 (1.350–67.137)*
TG > 1.7 mmol/L			
No	Ref.	Ref.	Ref.
Yes	0.339 (0.114–1.068)	0.416 (0.128–1.405)	0.231 (0.033–3.262)
TC > 5.2 mmol/L			
No	Ref.	Ref.	Ref.
Yes	0.452 (0.200–1.102)	0.395 (0.160–1.315)	0.213 (0.025–3.325)
e GFR ≤ 60 ml/(min∙1.73 m^2^)			
No	Ref.	Ref.	Ref.
Yes	2.576 (1.370–6.038)*	3.402 (1.415–8.328)*	2.732 (1.248–16.348)*

All models included age, sex, smoking, obesity, hypertension, DM, PP, TG, TC, e GFR and hyperuricemia. *P <0.05 versus reference category. AF indicates atrial fibrillation; DM, diabetes mellitus; e GFR,estimated glomerular filtration rate; PP, pulse pressure; TC, total cholesterol; TG, triglycerides.

The optimal cutoff point for SUA predicting AF was 370.5 µmol/L in overall very elderly (p = 0.006), 370.5 µmol/L in very elderly with hypertension (p = 0.012) and 397.0 µmol/L in very elderly with DM (p = 0.077) according to the ROC curve. The sensitivity and specificity of SUA ≥370.5 µmol/L for predicting AF were 62.7% and 61.5% in overall very elderly, respectively.

## Discussion

The main findings in this community-based study are as follows: First, we found that the prevalence of AF in the community very elderly was relatively high. Second, hyperuricemia was associated with the prevalence of AF in general very elderly Chinese in Chengdu.

### AF prevalence

To our knowledge, in China, until now, there is not a large national-wide epidemiological survey focusing on AF prevalence^2^. Moreover, recent epidemiological studies on AF were more about either local epidemiological status of AF in population younger than 80 years old or in patients with special characteristics^2,12,13^. A recent study using in-hospital record data in Yunnan reported that AF prevalence in patients 80 years older was about 0.77%, which may only represent the prevalence in population received medical treatment^14^. Another study in Shanghai with more than 350 patients older than 80 years in a newly urbanized suburban town reported a similar AF prevalence with our study, which was about 5.9%^15^. Moreover, a survey including about 1000 elderly older than 75 years in eight Chinese longevity areas, reported that the AF prevalence in subjects older than 80 years was 8.8%, which was a little higher than the prevalence in our study^16^. The prevalence of AF in our study population was relatively lower than prevalences above in other studies. One possible reason is that our study subjects were the very elderly in community while not in hospital patients or subjects with specific diseases, therefore, this study should be more accurately representative of the real epidemiological situation in the general very elderly.

### Association between UA and AF

In this study, participants with AF had a higher SUA level compared with participants without AF. Moreover, the prevalence of AF in participants with hyperuricemia was significant higher than in those without hyperuricemia. And our study also demonstrated that hyperuricemia was a factor for AF not only in general very elderly but also in very elderly with hypertension and DM, which is in accordance with another study in patients with DM^17^.

High SUA level has been found to be associated with AF in patients with chronic systolic heart failure^11,18^. High SUA level was also associated with prevalent AF in hypertensive patients without significant comorbidities^10^, and the development of AF in elderly people with normal blood pressure^19^. Also, SUA level was significantly associated with AF in general Japanese population^20^. Our study further confirmed this association between hyperuricemia and AF, and added information about this association in very elderly population. Although the mechanism of association between hyperuricemia and AF is not well understood, studies have suggested that oxidative stress^21^,^22^ and inflammation^23,24^ are the most likely contributing factors for AF development. The increase of superoxide and its reactive metabolites through activation of xanthine oxidase may contribute to the pathological consequences of AF such as thrombosis, inflammation, and tissue remodeling^25^. Hyperuricemia could induce protein expression in cells, causing inflammation through activation of uric acid transporters^23,24^. Moreover, UA facilitates the activation of the renin-angiotensin-aldosterone system at the local level by stimulating cell proliferation and up-regulating the cellular expression of angiotensinogen, angiotensin-converting enzyme(ACE), and angiotensin II receptors and increased angiotensin II levels, which were ameliorated by ACE inhibitors, UA transporter blockers, and antioxidants^25,26^. Therefore, accumulation of UA inside atrial cardiomyocytes might cause atrial structural remodeling, which provides a vulnerable substrate for AF triggering mostly by either oxidative stress, inflammation, or both.

### Study limitations

There are several limitations in this study. First, this cross-sectional study could only reflect the associations between factors and AF. Second, AF is often asymptomatic and can be intermittent, therefore widely used rest ECG could not detect all paroxysmal AF. However, AF prevalence is similar to results of studies in other Chinese cites using the same AF detection method^15,16^. And third, our study could only describe epidemiological distribution in very elderly in Chengdu, therefore our findings may limit generalizability to other very elderly population in China or other regions.

The prevalence of AF is relatively frequent in this community very elderly. No significant sex difference was found in this study. Hyperuricemia is associated with the prevalence of AF in general community very elderly Chinese in Chengdu.

## Methods

### Study population

This study was designed as a cross-sectional study in general community very elderly (≥80 years old) in Chengdu^27^. From May 2013 to May 2015, a representative sample of very elderly men and women in Chengdu were recruited by using of a stratified three-stage cluster sampling design. A total of 1056 very elderly from 20 residential communities were sampled according to registration data from Chengdu government. Inclusion criteria were permanent residents of the households with a record in the household registration (by identity card or permanent residence booklet checking) and living in local communities at least 3 years. Exclusion criteria were participants with any secondary hypertension, severe frailty, neurologic and psychological diseases (dementia, Alzheimer’s disease or schizophrenia, etc.), and disabilities or other problems who could not fully participate in this survey. The study protocol conforms to the ethical guidelines of the 1975 Declaration of Helsinki as reflected in a prior approval by the Ethics Committee of the Second People’s Hospital of Chengdu. All participants gave informed consent.

### Data Collection and measurement

Data were collected during clinic visits by well trained physicians and nurses using a questionnaire-based face-to-face interview. Data on basic demographic characteristics, medical history, lifestyle risk factors were obtained from a standardized questionnaire during the interview with participating investigators.

Rest 12-lead electrocardiogram (ECG) (ECG-1350P, Nihon Kohden, Japan) for participants was performed by a well trained physician. ECGs were analyzed by two experienced cardiologists independently for the determination of AF, defined according to the 2011 ACC/AHA/ESC guidelines^28^. Atrial flutter, atrial tachycardia, other arrhythmia and abnormalities were also recorded, but only AF was included in the present analysis^28^. The disagreement of analysis results was checked by a third cardiologist.

Fasting blood samples were collected in the morning after at least 8 h of fasting for all participants. SUA and other biochemical parameters, such as fast glucose (FG), total cholesterol (TC), triglycerides (TG), and low–density lipoprotein cholesterol (LDL-C) were analyzed enzymatically on an auto-analyzer (AU5421 Chemistry Analyzer, Beckman, Brea, California, United States) in the central laboratory of the Second People’s Hospital of Chengdu.

Blood pressure (BP) were measured three times at two-minute intervals after at least 5 min of rest using a standardized automatic electronic sphygmomanometer (HEM-7300, Omron, Kyoto, Japan) according to the Chinese Guidelines for Prevention and Treatment of Patients with Hypertension^29^. Weight and height were also measured. The body mass index (BMI) was calculated as weight in kilograms divided by the square of the height in meters.

### Definitions

AF was diagnosed based on rest ECG findings (absence of consistent P waves, presence of rapid, irregular f waves with a frequency of 350–600 beats/min, and an irregular ventricular response).

Hyperuricemia was defined by the levels of SUA >416 µmol/L (7.0 mg/dL) for men and >357 µmol/L (6.0 mg/dl) for women or if they were on allopurinol therapy.

Hypertension was defined as systolic BP ≥140 mmHg and/or diastolic BP ≥90 mmHg and/or self-reported treatment of hypertension with antihypertensive medication (including diuretics) in the last 2 weeks according to the Chinese Guidelines on Prevention and Control of Hypertension^30^. Impaired fasting glucose (IFG) was diagnosed if 6.1 mmol/L ≤ FG <7.0 mmol/L. Diabetes mellitus (DM) was diagnosed if FG ≥7.0 mmol/L, or FG <7.0 mmol/L with a past history of DM^30^. Obesity was defined as body max index (BMI) at least 28.0 kg/m^2^ according to World Health Organization guidelines for the Asian Pacific population^31^.

### Statistical analysis

Analyses were performed using SPSS software (Version 17.0, SPSS Inc, Chicago, IL). Continuous variables are expressed as mean ± standard deviation, and their normality was checked by the Shapiro-Wilk test. Frequencies are presented as percentages with 95% confidence interval (95%CI). Statistical comparison of means between groups was conducted using Student’s t-test or Wilcoxon signed-rank test. And x^2^ test was applied to compare proportions. Stepwise multiple logistic regression models were used to evaluate the association between risk factors and AF. The receiver operating characteristic (ROC) curve analysis was used to evaluate the efficiency of SUA in predicting AF. A two-sided *P* value < 0.05 was considered statistically significant.
